# An Investigation on the Regenerative Effects of Intra Articular Injection of Co-Cultured Adipose Derived Stem Cells with Chondron for Treatment of Induced Osteoarthritis

**DOI:** 10.15171/apb.2018.035

**Published:** 2018-06-19

**Authors:** Sorayya Jacer, Hajar Shafaei, Jafar Soleimani Rad

**Affiliations:** ^1^Department of Anatomical Science, Tabriz University of Medical Sciences, Tabriz, Iran.; ^2^Stem Cell Research Center, Tabriz University of Medical Sciences, Tabriz, Iran.

**Keywords:** Adipose stem cell, Cell therapy, Chondron, Co-culture, Induced osteoarthritis, Chondron

## Abstract

***Purpose:*** Adipose tissue derived stem cells (ASCs) and chondrocytes are best cells for articular cartilage regeneration. Chondrocyte with peri-cellular matrix (PCM) is called chondron provides ideal microenviroment than chondrocytes. We aimed to evaluate the regenerative effects of intra-articular injection of ASCs co-cultures with chondron in induced osteoarthritis (OA).

***Methods:*** ASC, from the peri-renal fat of male rat and chondron from primary newborn rat hyaline cartilage were isolated. ASCs were cultured for at least three passages in vitro. Six weeks after OA induction, rats were randomly distributed in five groups of control, osteoarthritic, ASC, chondron and co-cultured. ASCs (10^7^), chondrons (10^7^) and combination of chondrons and ASCs (10^7^) were injected into intra-articular space of the rat knee. The effect of treatments was evaluated by macroscopic and microscopic examinations. The expression levels of collagen type ΙΙ was studied by immunohistochemistry.

***Results:*** Macroscopic appearance of the co-cultured group, showed much enhanced articular cartilage regeneration compared to ASC and chondron groups. H&E showed evidence of repair site of articular surface without erosion and fibrillation versus OA group which showed thin layer of hyaline cartilage over tidemark and spontaneous fibrocartilage formation. Metachromatic regions stained with toluidine blue were larger in treatment groups versus OA group. Strong intensity of type ΙΙ collagen staining was observed in co-culture group compared to other groups.

***Conclusion:*** Co-culture of chondrons and ASCs increased articular hyaline cartilage formation and provides a useful tool to improve limitations of each of applied cells in this model.

## Introduction

Osteoarthritis (OA) as a degenerative multifactorial joint disease is the most frequent form of the musculoskeletal diseases in the world.^1^ The number of patients afflicted with OA is expected to rise in elderly population and it will impose remarkable financial and social burdens consequently it can affect quality of life in many aspects.^2^ Hyaline cartilage tissue has‏ less intrinsic regenerative capacity due to‏ the absence of vascularity‏. In fact, a major challenge for orthopedists surgeons‏ is the lack of efficient treatment to repair‏ of large chondral lesions. Consequently, it has prompted investigations on tissue engineering along with chondrogenic cells.^3^ In 1987, Brittberg *et al*, represented cell therapy as a treatment for patients suffering of knee articular cartilage‏ defects.^4^ For cartilage tissue regeneration the use of three key elements included of chondroprogenitor cells source, biocompatible scaffold and bio-substances as growth factor is necessary.^5^ The first introduced technique to repair focal cartilage defects was autologous chondrocyte transplantation (ACT).^6^ ACT method‏ is‏ accompanied with damage of uninvolved areas of joint cartilage and second surgical step for re-implantation of expanded chondrocytes‏ into the areas of the chondral injuries.^7,8^ In addition, based on the available data reported by Roberts and coworkers‏ ACT model is an expensive and time-consuming method compared to other recent methods.^9^ Adult mesenchymal stem cells (MSCs) are attractive cell population versus chondrocytes for cell based treatment strategies.^7^ Recently, much of attention has been focused on the therapeutic potential of MSCs due to their capacity for self-renewal and anti-inflammatory properties for cell-based cartilage repair.^4,5,10-12^ Adipose-derived stem cells (ASCs) particularly are believed to be applicable cell sources as they can be simply harvested in large scale with less donor site morbidity.^13^ The most of studies about cell based treatment strategies were conducted using bone marrow derived MSCs^14-20^ and some information are available on ASCs applications in cartilage repair methods.^21,22^ Some previous studies have reported expression of cartilage hypertrophy markers such as collagen type X and an increase in alkaline phosphatase activity by MSCs undergoing chondrogenesis in a pellet culture system.^14,23^ Additionally, the obtained data from the most‏ of animal model systems represented mineralization and systematic vascular invasion after ectopic transplantation of *in vitro* expanded MSCs.^23^ Hence, the expression of hypertrophic markers indicates an undesirable chondrogenesis due to inappropriate induction conditions and ultimately resulting in endochondral ossification.^24,25^ To date, several remarkable locally produced factors such as Bone Morphogenetic Proteins (BMPs), Fibroblast Growth Factors (FGFs), Transforming Growth Factor Beta (TGFβ), Parathyroid Hormone-Related Peptide and Retinoid are known to induce cartilage hypertrophy.^26^ Based on this information, researches are challenging to find appropriate chondrogenic induction factor. Recently, co-cultures of articular chondrocytes and MSCs have been proposed to eliminate the mentioned challenges. Tsuchiya *et al* introduced co-culture of chondrocytes with MSCs.^27^ According to some proceeding evidences from several studies, the interactions between MSCs and chondrocytes showed great potential in effective chondrogenesis in cartilage regeneration.^27,28^ The other co-culture experiment‏ has reported that OA chondrocytes can enhance chondrogenic differentiation of human MSCs.^29^ Further studies by Vinatier^30^ and Pittenger and their co-workers^31^ suggested that co-culture of human embryonic stem cells and human articular chondrocytes results in significantly altered phenotype and improved chondrogenic differentiation. Also, Bigdeli *et al* demonstrated that co-culture of MSCs with human articular chondrocytes help to articular chondrocytes differentiation.^32^ Plenty of increasing experiments have investigated the effect of chondrocyte-secreted morphogens from normal chondrocytes on chondrogenic differentiation of stem cells.^33-36^

It can properly maintain‏ chondrocyte phenotype when peri-cellular matrix (PCM) of chondrocyte is preserved.^37^ Chondrocytes are embedded within an immediate narrow PCM together termed as chondron.^38^ The PCM consisted of Hyaluronan, proteoglycans, glycosaminoglycans, and collagen type II and IV for attachment of chondrocyte to the PCM.^39^ The protocol of enzymatic chondron isolation introduced for the first time by Lee *et al* to reach high amounts of viable chondrons for biological and biochemical characterization.^40^ Subsequently, chondrons have demonstrated distinctive characteristic from isolated chondrocytes due to the retention of interactions between the PCM and chondrocytes.^40-43^ Preservation of the PCM in chondron leads to conservation of the metabolic activity in chondrocytes, and help to regulate gene expression and the growth factors production.^41,44^ In a co culture study on freshly cartilage defects in goats it has been shown that cell combination of chondron and MSC produce higher level of glycosaminoglycan and extra cellular matrix extraction in comparison with co culture chondrocyte and MSC of freshly created cartilage defects in goats.^45^ Owida *et al* in *in vitro* study represented co-culture of chondrons with MSC reduces the loss of collagen VI and improves extracellular matrix production of chondrons.^46^ It is possible that the combination of chondron with ASCs can repair cartilage lesions rather than chondrocyte in *in vivo*. In addition, limitations related to source and number of chondrocyte will be eliminated using stem cells. Moreover ASCs cells release several anti-inflammatory and immunomodulatory factors.^47^ Therefore, accessibility, abundancy and immunomodulatory properties of ASC make it an invaluable cell type for the repair of cartilage damages such as OA as an inflammatory disease. Therefore, this study aimed to investigate the regenerative effects of ASCs co culture with chondron in induced osteoarthritis model in the knee joint of rat.

## Materials & Methods

Six male new-born rats were assigned for chondron isolation, seventeen (2 rats for ASC isolation, and 15 rats for experimental groups) mature male rats. The rats were housed in groups of 2 per plastic cage in a 12:12 light-dark cycle with controlled temperature. They were fed a standard diet and were provided access to filtered standard water.

### Isolation of Adipose-derived Mesenchymal Stem Cells

Initially, the adipose tissue was isolated from the peri-renal adipose tissue of mature male rat under sterile conditions and transferred to a 50-mL sterile falcon tube containing Phosphate-Buffered Saline (Biosera, XC-S2067) and 1% Penicillin-Streptomycin (Sigma, P0781). The harvested adipose tissue minced into small fragments approximately 2×2×2 mm. small pieces of adipose tissue were washed with PBS several times then were subjected to collagenase I enzyme (Worthington, 4154) (0.5 mg/ml) for each gram of tissue with gentle shaking at 37°C for 1hr. The digestion was terminated with DMEM supplemented with 10% FBS and 1% Penicillin-Streptomycin. Subsequently, cell suspension was centrifuged at 1600 rpm (Orum tajhiz, Iran) for 10 minutes. At this step, the cell pellet re-suspended and seeded on culture flask medium containing DMEM (Gibco, 32500-035) 10% FBS (Gibco, 10270-106), 1% penicillin-streptomycin cultured in the incubator (37ºC, 97% humidity, 5% CO2). Following 24 h, the culture medium was washed with PBS to remove erythrocytes and detached cells. Two or three days later the culture medium was replaced. After reaching 80% confluence, the cells were treated with %0.25 Trypsin and 0.02 mM EDTA (Gibco, 25200-056) solution and the cells reseeded under the same conditions for expansion as passage 1.^48^

### Chondron extraction

Cartilage samples were isolated according to previously described methods and were prepared from newborn rat's limb hyaline cartilage. Briefly, the cartilage pieces were rinsed 3 times with PBS containing 1% Penicillin-Streptomycin. Then cartilage tissues were minced to smaller pieces. The harvested fragments were subjected to 0.2 % collagenase type II in PBS at 37° C with agitation for 5 h and mechanical isolation.^40^ The medium containing (10% fetal bovine serum and 1% Penicillin-Streptomycin was added to block enzymatic digestion. After centrifugation at 1600 rpm for 10 minutes the supernatant, was discarded and cell pellet was prepared separately for chondron and co-culture combination of cells.

### Induction of rat Osteoarthritis 

In this step, the adult male rats weighing (250-300 g) were anesthetized under ketamine (Rotex Medica) and Xylazine (Alfasan) anaesthetic combinations (60 mg/kg I.P, and 10 mg/kg I.P, respectively).^49^ Osteoarthritis was induced by intra-articular injection of 20μl of collagenase II(Gibco, USA) (90 mg/ml) into the knee joint of rats through the patellar ligament with the use of an insulin syringe.^50,51^ To provide pain relief oral dose‏ of 300 mg/kg of acetaminophen (Rouz Darou) dissolved in drink water was administered for rats.^52^

### Co-culture of ASCs and Chondrons

Harvested chondrons were seeded into 6-well plates for 24 hrs. The unattached chondrons on culture dish are injected for CHN group (10^7^) and the rest of floating chondrons (5x10^6^) were added to ASCs monolayer cultures. After 24 hrs, chondrons are removed by medium and ASCs are trypsinized. Then, combination of each cells (5x10^6^) are injected for co-culture group.

### Intra-articular injection ASCs and chondrons

The rats without OA considered as control. Six weeks after induction of OA, rats were randomly distributed in four groups. In the groups (OA) or Osteoarthritic control (no cells, only injection of 20 μl PBS). Other were assigned as experimental group (ASC) with injection of ASCs (10^7^) in 20 μl PBS, the experimental group (CHN) with injection of 20 μl of chondron cell suspension (10^7^) and finally co-culture group with injection of 20 μl ASC and chondron cell suspension (10^7^).

### Macroscopic examination 

Rats were sacrificed at 3 months after intra-articular injection of cells by chloroform anaesthesia. Then knee joints were dissected and the surfaces of the cartilage of the femoral condyle and tibial plateau were observed under loop microscope by two observers who were blinded to treatments. Samples were processed and sectioned for histopathological examination.

### Histologic assessment 

Sagital sections of knee were fixed with fixative solution (10% neutral-buffered formalin) within 48 hours. Then decalcification was carried out after the specimen has been thoroughly fixed to remove calcium deposits by aqueous 5% nitric acid. Decalcifying solution was replaced daily till the tissue soft enough to be cut by microtome and then washed with distilled water.^53,54^ The decalcified specimens were then routinely processed. In this procedure, tissues were dehydrated through a series of graded ethanol baths to displace the water. Subsequent removal of de-hydrant was carried out by immersing the specimens in xylene as clearing agent. To create of a permanent block of tissue the samples were then infiltrated with paraffin. Infiltrated tissues were then embedded into paraffin blocks, and cut into the desired thickness with vertical plane of 5 μm sections using a microtome (Leica, Germany). Finally, histological sections were stained with Haematoxylin/ Eosin (H&E) and Toluidine blue.

### Toluidine Blue Staining

Sections of tissues were stained with cationic toluidine blue to facilitate identification of proteoglycans components. The next step was clearing in which slides were immersed in xylene for 30 min (repeated 3 times) until the paraffin has been dissolved. Then samples were hydrated by passing the slides through graded alcoholic solutions decreasing ethanol concentrations of 96, 96, and 70% (2 times in 96% and after 1 time in 70% alchole), 1 minutes in each. To remove any deposit tissues were washed in double distilled water for 1 minute. The sections were flooded with 0.1% aqueous Toluidine blue and incubated for 1–10 minutes then were washed with water for 1 minute and slides were left to air dry for 9 minutes for immune-staining the slides treated for about 5 minutes in xylene. Mounting media (resin) was then placed on to the sections, then the coverslips were applied and firmly pressed down to be ready for examination.^55^

### Immunohistochemical staining method

To perform the immunohistochemical (IHC) staining, sections of 3-5 microns were prepared from tissues and placed on the coated slides with poly L-lysine (Sigma, P8920) and dried overnight at room temperature. To perform antibody staining, paraffin wax removed from the samples and the samples were rehydrated. Then 10 μg/ml of proteinase K (YT, 9052) were applied as retrieval antigen and the slides were washed in running tap water for 5 minutes. In order to neutralize the activity of the tissue peroxidase, the slides were exposed to hydrogen peroxide (to prepare 100 ml, add 10 ml 30% hydrogen peroxide to 90 ml H2O) in distilled water for 5 minutes. After washing, sections were transferred to a Tris-Buffered Saline (Merk). The sections were incubated in blocking buffer containing primary anti-collagen ΙΙ anti-body (1:100 ab3092, US) at 4°C overnight. The next day, the sections were placed at room temperature for 30-40 minutes before washing with TBS. The incubation was then performed using 100 μl of secondary antibody (1:100 AP8036, RAZI Bio Tech) at 4 °C for 30-60 minutes in a humidified chamber. The enzyme horseradish peroxidase (HRP) was used as a convenient tracer to catalyse the conversion of colourless chromogenic substrates (DAB Chromogen) into a brown substance seen in the microscope. The resulted slides were washed with water for 10 minutes and brown-colored DAB reaction products were visualized by light microscopy. At the end the sections, stained with Haematoxylin.

## Results

### Macroscopic findings

#### Cell injection repairs cartilage erosions of OA 

**Figure 1:**
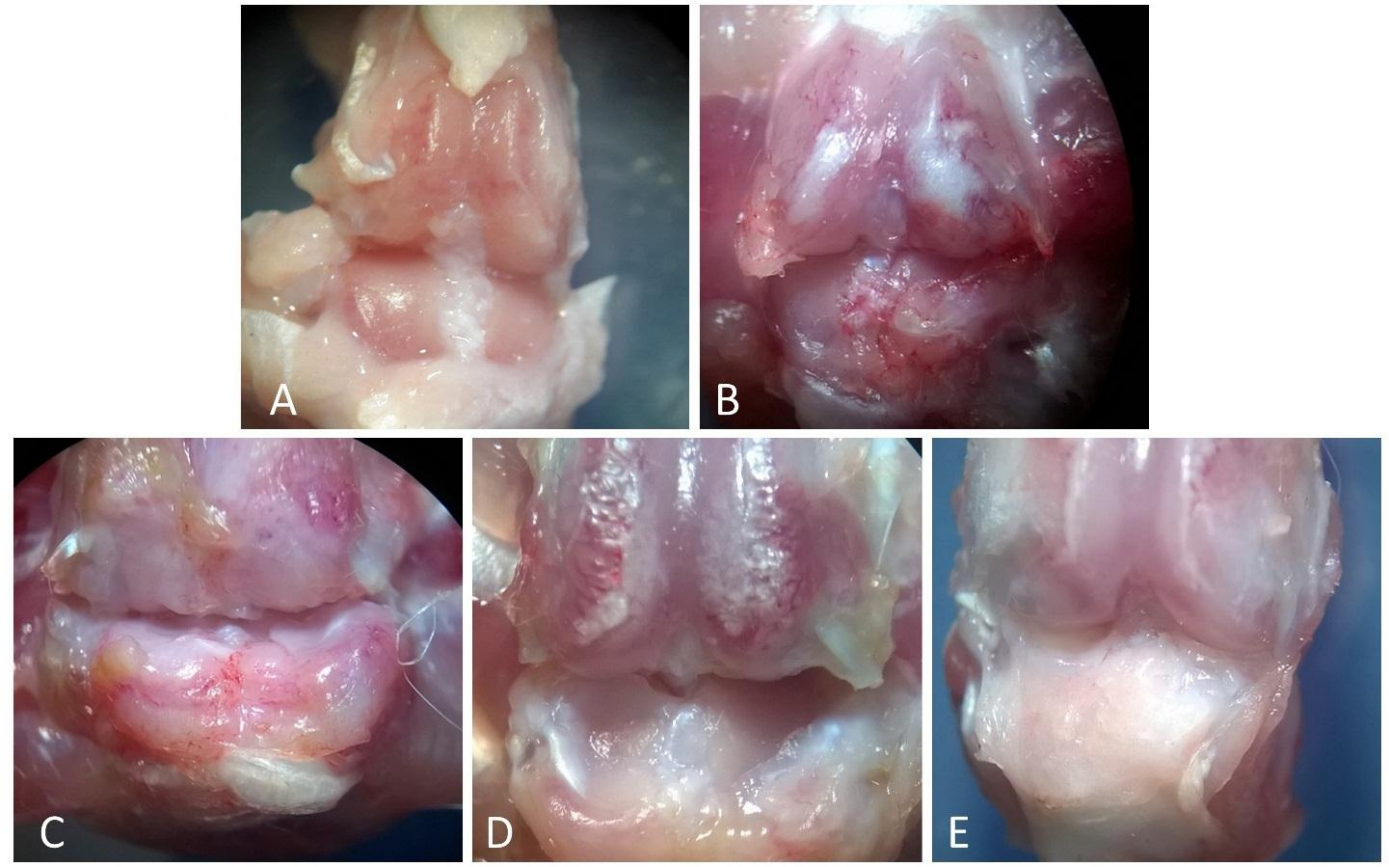
Gross morphological observation of articular cartilage in the knees of the rat, Magnification: X10. A) The gross appearance of control group with smooth surface with no osteophytes or erosions, B) The osteoarthritis group: erosion was observed in joint surface, C) The ASC group. Partial regeneration of erosion in articular surface compared with OA group, D) Chondron group. Regeneration of erosion in articular surface compared with OA group, E) Co-culture of chondron and ASC group: Smooth articular surface with no erosions.

The isolated articular cartilage surfaces from 5 treatment groups were scored ‘blind’ in code by two readers. There was no gross evidence of any side effects such as infection or tumor formation throughout the observation period. The cartilage surfaces from the rats in the OA group exhibited moderate erosion compared with control group (Figure 1A and 1B). Also, macroscopic observations from femoral condyle in ASC group represented grossly evident of less articular cartilage erosion and fibrillation in most regions of the articular knee surface compared with the OA group (Figure 1C) Macroscopically, in the co-cultured (Figure 1E) group cartilage regeneration was significantly higher versus all other groups whereas no significant regeneration was found in chondron group (Figure 1D) in comparison to other different treatment groups.

#### Microscopic findings

#### New hyaline cartilage tissue formation on osteoarthritic articular cartilage by intraarticular injection of cells 

**Figure 2:**
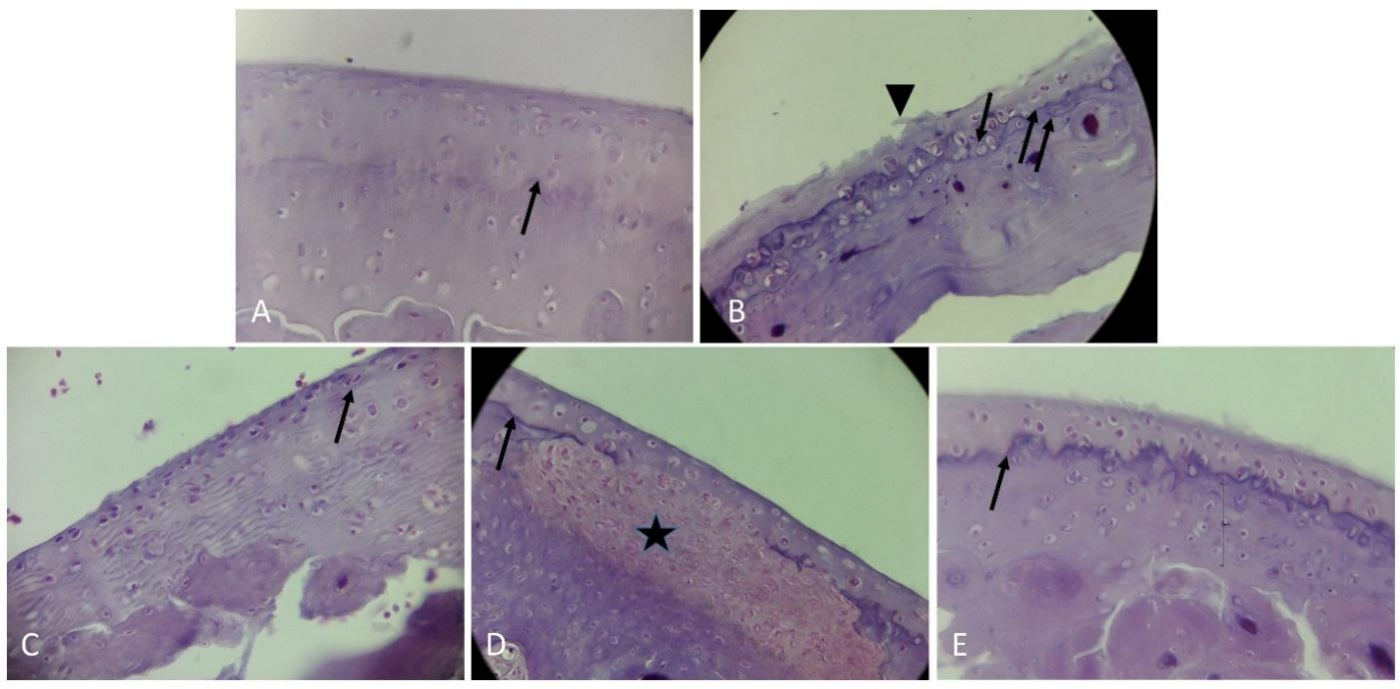
Photomicrographs of sagittal sections showing articular knee joint of rats, stained with Hematoxylin-Eosin, magnification: X400. A) The control group with smooth articular surface consisted of small and stretched superficial cells in tangential zone, transitional zone and the deeper zone cells were arranged perpendicular to the articular surface. The narrow arrow represents Tidemark line, B) The OA group with microscopic signs of osteoarthritis and reduction of knee hyaline cartilage thickness. Arrows denote the histologic duplications or disappearance of the tidemark line and superficial fibrillation that designated with head arrows, C) The ASC group. Showing hypercellularity and marked increase in numbers of superficial chondrocytes compared with control group, D) Chondron group. Self-regeneration of erosion in articular surface with mass of hypercelluar tissue which star symbol indicates a self-healing area under the newly formed hyaline cartilage, E) Co-culture of chondron and ASC group: The cartilage regeneration is observed but the thickness of the hyaline cartilage is less than that of the calcified region.

Articular cartilage alterations in H&E staining: In our study, we evaluated contour and surface quality changes of articular cartilage in the determined groups. In the control group morphology of articular cartilage was apparent by smooth surface and flattened superficial chondrocytes. Spindle-shaped cells of tangential zone, rounded cells of transitional zone and the deep zone cells were arranged perpendicular to the articular surface. Tidemark line (a visible basophilic line that separates deep, and calcified zones) was distinctly evident in our observations. Besides calcified cartilage was seen at the interface between tidemark line and the subchondral bone plate (Figure 2A). In this figure the narrow arrow represents Tidemark line. In histopathological examination for OA group at the site of progressive degeneration of articular cartilage decreased cartilage thickness, increased roughness of articular surface and loss of tidemark integrity were observed. In addition, OA group in regions with limited joint cartilage destruction tidemark duplications was directly accompanied with fibrillation on the surface of articular cartilage (Figure 2B). Spontaneous repair of cartilage was observed by formation of fibrocartilage tissue with high fiber and low cell density. In the OA group, Sub-Tide mark calcified layers of cartilage was greater than control group. Chondrocytes were smaller and oriented in parallel longitudinal axis. Arrows denote the histologic duplications or disappearance of the tidemark line and superficial fibrillation that designated with head arrows (Figure 2B).

Obviously, in the ASC group, the population of chondrocytes was higher in comparison to OA group (Figure 2C). In the ASC group, spindle shaped superficial cells were seen. Also, chondrocyte lacunas were smaller than that of control group. While in the chondron group on the site of the cartilaginous lesions, visible hyaline cartilage chondrocytes with rounded morphology were detected (Figure 2D). Unlike ASC group, in the chondron group the hyaline cartilage was localized on the top of cartilage lesions and regenerative chondrocytes exhibited a rounded shape morphology. Unfortunately, due to the impossibility for tracing of mentioned cells in our study the cellular origin these cells cannot be identified (Figure 2D). In Figure 2D star symbol indicates a self-healing noncartilaginous tissue under the newly formed hyaline cartilage.

Also, apparently in co cultured group‏ one layer of injected cells were located on self -regenerated hyaline cartilage layer (Figure 2E). Study of the subchondral bone structure region by observers represented a thinner subchondral bone plate in OA group in comparison with control group. Our data showed that treatments including ASC, chondron and both of them had no effect on subchondral bone plate thickness (Figure 2).

#### Toluidine blue staining findings

**Figure 3:**
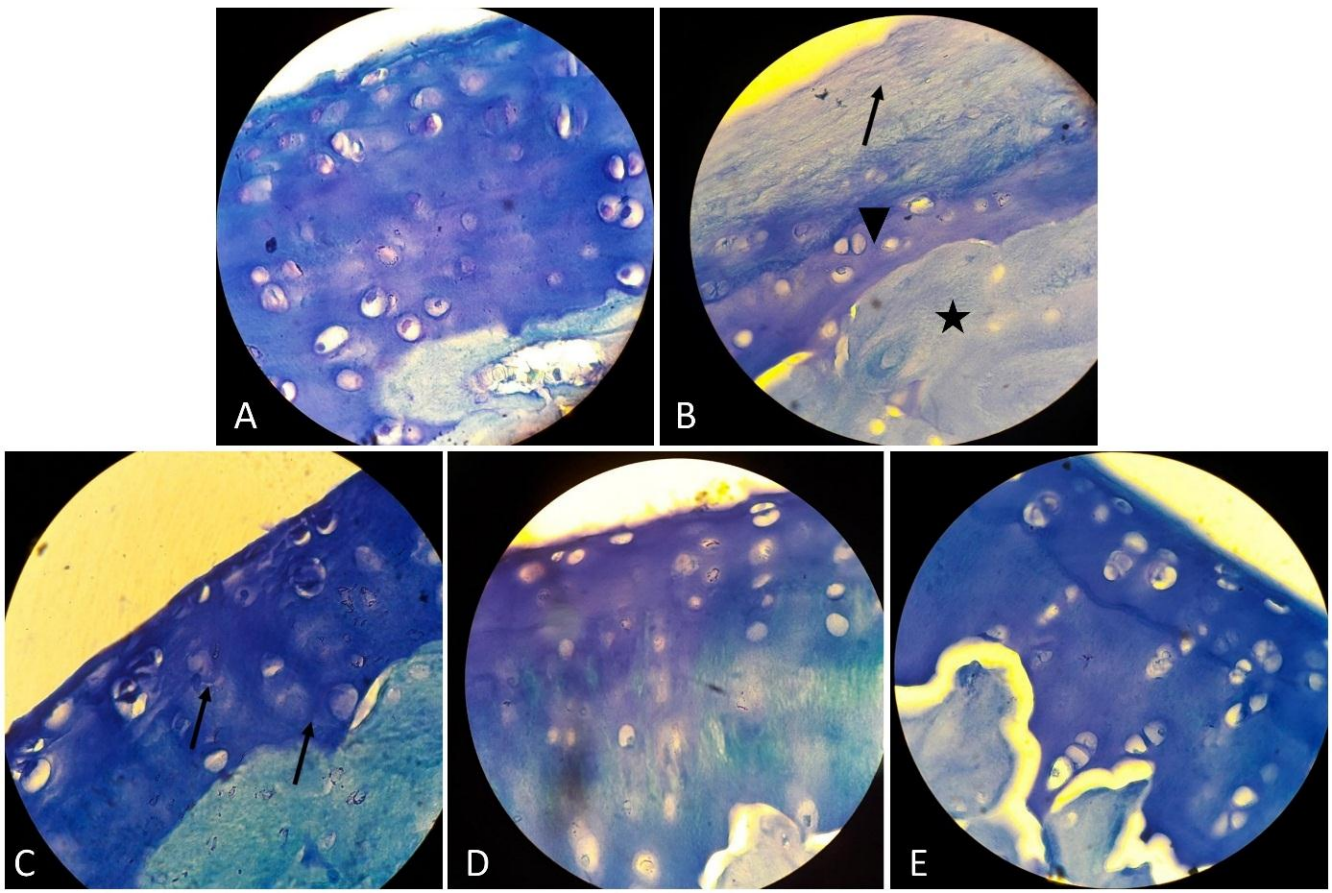
Evaluation of proteoglycan content (glycosaminoglycan) of articular cartilage by Toluidine- Blue (TB) staining –magnification: X1000; A) The control group: Metachromatic staining reaction in proteoglycan component is indicative of the presence of GAG in the hyaline cartilage matrix, B) The OA group: The absence of metachromasia is due to reduction of GAG, which indicates structural changes. The arrow, arrow head and star symbols, are respectively hallmark of the formation of a self- renewed fibrouse, cartilage and bone tissues, C) The ASC group. The arrows show The metachromatic regions around the cells that are indicative of renovation of GAG, D) Chondron group. The metachromatic areas around the cells represent the production of glycosaminoglycan, E) Co-culture of chondron and ASC group. Normal hyaline cartilage with less total glycosaminoglycan content compared to the control group.

To evaluate the proteoglycan content (glycosaminoglycan) of articular cartilage in the studied groups, Toluidine blue staining was performed. In the control group, metachromasia of ECM was seen which was due to the presence of many glycosaminoglycan components around the chondrocytes Figure 3A. In the OA group, there is no metachromatic region in ECM but a layer of blue colour was seen which was histological hallmark of destruction of articular cartilage and the formation of a fibrocartilage layer (Figure 3B). The arrow, arrow head and sign star symbols in Figure 3B, are respectively hallmark of the formation of a self- renewed fibrous cartilage and bone tissues in the OA group. In the ASC group (Figure 3C) the arrows show the metachromatic regions around the cells that are indicative of renovation of GAG. In the chondron group the metachromatic areas around the cells represent the production of glycosaminoglycan (Figure 3D). In the co-culture of ASC and chondron group normal hyaline cartilage with less total glycosaminoglycan content was seen compared to the control group (Figure 3E). 

#### Immunohistochemical findings

**Figure 4:**
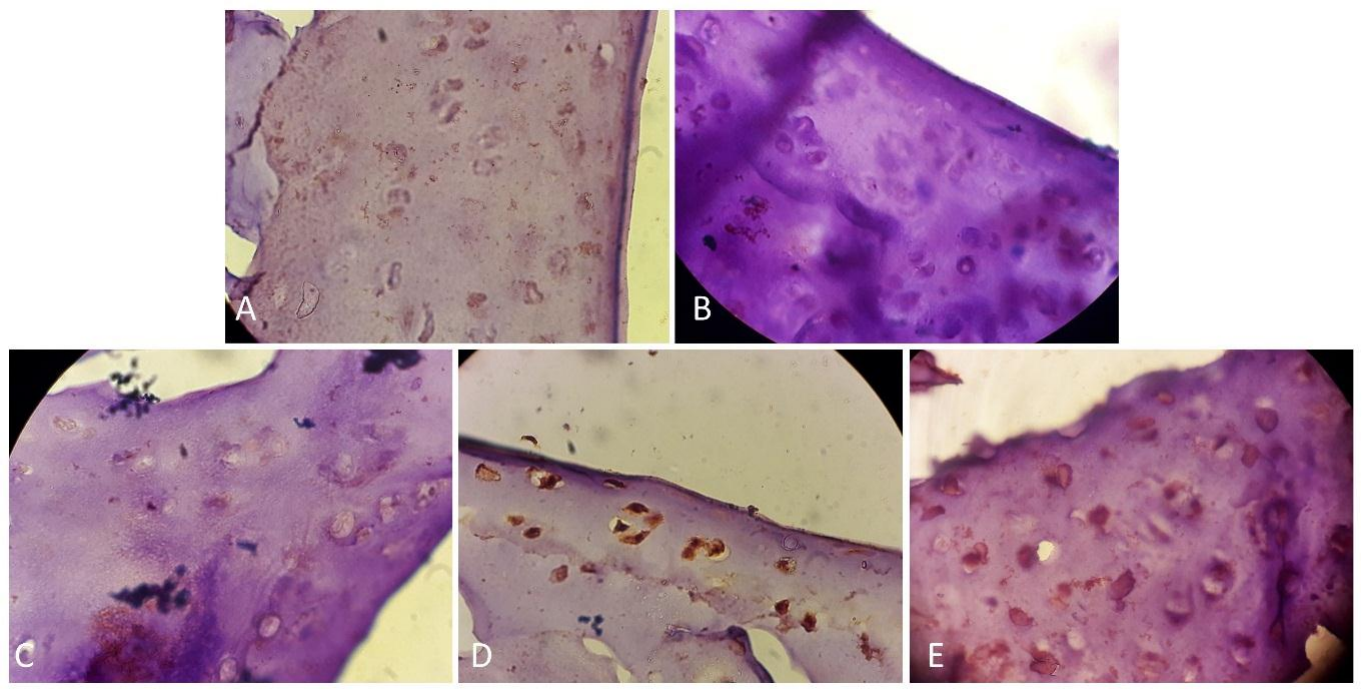
Immunohistochemistry staining for type II collagen in the articular cartilage at 12 weeks after the treatment. magnification of X1000; A) Control group: Positive collage type II staining (brown) was seen in matrix and particularly Pri Cellular Matrix, B) OA group: Immuno-histochemical staining revealed the absence or trace quantity of type II collagen in cartilage matrix, C) The ADSC group. The expression of type II collagen in the ASC treated group. D) Chondron group. The expression of type II collagen in the chondron treated group, E) Co-culture of chondron and ASC group: Section area exhibiting higher expression for type ΙI collagen in co-culture of chondron and ASC group in comparison to chondron and the ASC group.

The expression levels of collagen ΙΙ were studied by immunohistochemistry method. Strong intensity of staining was indicated to immune-positive reaction for collagen type ΙΙ in control group (Figure 4A). In OA group, expression levels of type ΙΙ collagen were prominently decreased compared to the control group (Figure 4B). Overall, here immune- histochemical findings within treated groups of the ASC (Figure 4C) and chondron groups (Figure 4D) showed highly elevated levels of collagen type ΙΙ as compared to OA group. In the co-cultured group expression levels of collagen ΙΙ was higher versus all treated groups (Figure 4E).

## Discussion

In articular cartilage regeneration, major limitations for cell therapy approaches are lack of chondrocytes source and inefficient differentiation of MSCs. This study aimed to employ co-culture system as a potent technique in tissue engineering to overcome these limitations. Our results on rat OA model showed that co-culture of chondrons and ASCs can be able to produce cartilaginous tissue with round chondrocytes in lacuna in comparison to ASC injection separately. The other limitation of chondrocyte transplantation is their dedifferentiation during *in vitro* expansion. Therefore, intra-articular injection of chondron in induced OA showed therapeutic effect of it.

In advantages of chondron against chondrocyte, Poole *et al* in a long-term investigation carried out an extensive body of experiments on chondron to identify the potential function of the PCM compartment.^41^ Furthermore, a large quantity of studies evaluated the PCM to recognition of its biochemical and mechanical properties.^37,41,42^ Another study made evident that newly formed repair cartilage tissue originated of chondrons along with PCM is the preferred choice for cartilage tissue engineering and had superior quality in comparison to chondrocytes. Of these, it has been shown that ECM has a major effect on gene expression and response to growth factors.^37^ Also, one goat model new study has shown that human chondrons produce more cartilage ECM than human chondrocytes, when cultured with human MSCs.^45^ Vonk *et al* have suggested that constructed cartilage tissue with chondrons contains higher amount of collagen and proteoglycan over a period of one month. Also they have suggested that a fully developed PCM is required for cartilage tissue engineering.^37^ Furthermore, their results suggest that the high level of collagen type ΙΙ and low level of collagen type Ι gene expression in the chondron produces more cartilage-like extracellular matrix than chondrocytes. Another study demonstrates that PCM structure has a profound effect on chondrocyte gene expression.^56^ Higher proteoglycan retention by chondron shows the importance of the PCM in the accumulation of the extracellular matrix.^57^ In agreement with our results chondrons produce better hyaline cartilage compared to chondrocytes.^37,38,58^ Therefore, chondron is considered as an ideal cell source for cartilage tissue engineering especially in co-culture systems with MSCs.

Adipose tissue has a broad range of clinical applications and has shown promise in the field of cartilage engineering as an abundant source of mesenchymal stem cells. During the past decade, numerous studies have provided preclinical data on the safety and efficacy of ASCs.^25,59^ The utilization of ASCs in cartilage tissue engineering is a rapidly growing area of research and some evidence of therapeutic achievement using ASCs for OA defect has been reported.^48,60,61^ In general, there have been fewer investigations on co-culture of chondrons with mesenchymal stromal cells (MSCs) in *in-vivo* systems.^62^ These studies are dividable in two categories. First categories showed that chondro-inductive effects of chondrocytes on MSC would provide chondrogenesis of MSC without inducing hypertrophy. It is based on that chondrons likely have the positive effect on MSCs by the PCM and second categories are based on that MSCs have the potential capacity to preserve the PCM of chondrocyte and improve the deposition of ECM in a dose response manner.^46,62,63^ Generally, in second categories, it has been shown that the chondron pellet co-culture with MSC make more glycosaminoglycan contents than chondron solely.^44^

These data suggest that chondrons together with MSC improved ECM production and demonstrated ability to maintain the PCM.^46^ In addition, one study strongly demonstrated that the levels of mRNA and protein expression of SOX9, COL2 and‏ Aggrecan in co-culture group of MSCs and direct cell-cell contact with Chondrocytes compared with indirect cell–cell contact between articular chondrocytes (ACs) and MSCs and also compared to chondrocytes and stem cells in separate culture conditions, have been increased.^64^ Sox9 potentiates BMP2-induced chondrogenic differentiation and inhibited endochondral ossification. Pervious data suggest that their results from co-culturing of half and half chondrons and MSC resulted in the most significant increase in GAG production, ECM synthesis and the preservation of the PCM. They demonstrated that co-culture of chondrons and MSCs reduces the loss of collagen VI and improves ECM production.^46^

## Conclusion

In conclusion, the current available study suggests that co-cultures using ASCs and chondrocytes with their PCM are able to produce cartilaginous tissue *in-vivo*. In this study, the effect of co-culture of ASCs and chondrons cells was only evidenced increased articular hyaline cartilage formation on injured area, and further studies are needed to evaluate phatways of repair, the efficacy and long-term safety of this system for the treatment of OA in human.

## Acknowledgments

Authors indebted to the research vice chancellor of Tabriz University of Medical sciences for providing grant for this research. The ethical code of this research is 95/2-7/4 from the ethic Committee of Tabriz University of Medical sciences.

## Ethical Issues

This study was performed under rules of the ethical committee of Tabriz University of Medical Sciences.
